# Closing the COVID-19 Psychological Treatment Gap for Cancer Patients in Alberta: Protocol for the Implementation and Evaluation of Text4Hope-Cancer Care

**DOI:** 10.2196/20240

**Published:** 2020-08-12

**Authors:** Vincent Israel Opoku Agyapong, Marianne Hrabok, Reham Shalaby, Kelly Mrklas, Wesley Vuong, April Gusnowski, Shireen Surood, Andrew James Greenshaw, Nnamdi Nkire

**Affiliations:** 1 Department of Psychiatry Faculty of Medicine and Dentistry University of Alberta Edmonton, AB Canada; 2 Addiction and Mental Health Alberta Health Services Edmonton, AB Canada; 3 Cumming School of Medicine University of Calgary Calgary, AB Canada; 4 Department of Community Health Sciences Cumming School of Medicine University of Calgary Edmonton, AB Canada; 5 Strategic Clinical Networks Provincial Clinical Excellence Alberta Health Services Calgary, AB Canada; 6 APEC Digital Hub for Mental Health Edmonton, AB Canada

**Keywords:** cancer care, COVID-19, pandemic, mobile phones, text messaging, anxiety, depression, e-mental health

## Abstract

**Background:**

Cancer diagnoses and treatments usually engender significant anxiety and depressive symptoms in patients, close relatives, and caregivers. Providing psychological support during the coronavirus disease (COVID-19) pandemic presents additional challenges due to self-isolation and social or physical distancing measures in place to limit viral spread. This protocol describes the use of text messaging (Text4Hope-Cancer Care) as a convenient, cost-effective, and accessible population-level mental health intervention. As demonstrated in previous research, this evidence-based program supports good outcomes and high user satisfaction.

**Objective:**

We will implement daily supportive text messaging as a way of reducing and managing anxiety and depression related to cancer diagnosis and treatment in Alberta, Canada. Prevalence of anxiety and depressive symptoms, their demographic correlates, and Text4Hope-Cancer Care–induced changes in anxiety and depression will be evaluated.

**Methods:**

Alberta residents with a cancer diagnosis and the close relatives of those dealing with a cancer diagnosis can self-subscribe to the Text4Hope-Cancer Care program by texting “CancerCare” to a dedicated text number. Self-administered, anonymous, online questionnaires will be used to assess anxiety and depressive symptoms using the Hospital Anxiety and Depression Scale (HADS). Data will be collected at onset from individuals receiving text messages, and at the mid- and endpoints of the program (ie, at 6 and 12 weeks, respectively). Data will be analyzed with parametric and nonparametric statistics for primary outcomes (ie, anxiety and depressive symptoms) and usage metrics, including the number of subscribers and user satisfaction. In addition, data mining and machine learning analysis will focus on determining subscriber characteristics that predict high levels of symptoms of mental disorders, and may subsequently predict changes in those measures in response to the Text4Hope-Cancer Care program.

**Results:**

The first research stage, which was completed in April 2020, involved the creation and review of the supportive text messages and uploading of messages into a web-based text messaging service. The second stage, involving the launch of the Text4Hope-Cancer Care program, occurred in May 2020.

**Conclusions:**

Text4Hope-Cancer Care has the potential to provide key information regarding the prevalence rates of anxiety and depressive symptoms in patients diagnosed or receiving care for cancer and their caregivers. The study will generate demographic correlates of anxiety and depression, and outcome data related to this scalable, population-level intervention. Information from this study will be valuable for health care practitioners working in cancer care and may help inform policy and decision making regarding psychological interventions for cancer care.

**International Registered Report Identifier (IRRID):**

PRR1-10.2196/20240

## Introduction

### Background

Cancer is a leading aspect of the global burden of disease [1] and the second leading cause of death internationally [2]. In 2018, 1 in 6 deaths worldwide (9.6 million deaths) were attributed to cancer [2]. The total annual economic cost of cancer in 2010 was estimated to be approximately USD $1.16 trillion, and the economic impact of cancer is significant and increasing [3]. The same trend exists in Canada, where cancer is the leading cause of death, responsible for about 30% of deaths [2]. Based on 2015 estimates, 1 in 2 Canadians are expected to develop cancer during their lifetime and 1 in 4 are expected to die from it; current figures estimate 225,800 new cancer cases and 83,300 deaths in 2020 [4]. In Alberta, about 1 in 2 Albertans will reportedly develop cancer, with 20,473 anticipated cases [5]. Furthermore, mental illnesses are the most prevalent disabilities in Canada, generating at least 70% of documented costs with a cumulative annual economic impact of approximately CAD $8 billion in direct costs (ie, hospital and physician visits and medications), and CAD $11-50 billion in indirect costs [6].

A new cancer diagnosis places significant emotional strain on individuals and their caregivers and is associated with an increased risk for common psychiatric disorders [7]. After cancer diagnosis, emphasis is necessarily placed on physical treatment, with limited attention on associated psychological complications [8].

The prevalence of anxiety and depression amongst patients with cancer varies; mean depression prevalence estimate from diagnostic interviews is 13% among cancer patients, with a range from 4%-49% depending on context and assessment methods [9]. An estimated 20%-30% of diagnosed patients develop depression and anxiety [10,11]. Patients with incurable disease report higher levels of anxiety and depression, compared with patients who can be cured [12]. Depression and anxiety may adversely impact treatment and outcomes for patients with cancer [7,8,13]. For patients with cancer diagnoses that exhibit poorer outcomes, such as lung cancer, the risk of suicide is higher than in the general population, and more so within the first 6 months postdiagnosis [14,15]. Patients who previously accessed mental health services are at increased risk of poorer outcomes, including greater risk of mortality after a cancer diagnosis [16]. As such, early assessment and treatment of these mental health conditions may produce a beneficial effect, particularly with respect to anxiety symptoms [2,17,18].

While determinants of the development of psychological distress or mental illness in cancer patients are not well understood [8,19], several factors have been proposed, including age, gender, lack of social support, unemployment, and fewer educational qualifications [8,20]. Several factors related to the cancer itself may also lead to the development of mental illness, including the type and stage of cancer, prognosis, and treatments offered and received [8]. Stigma arising from certain types of cancers (eg, lung cancer and tobacco use) and associated guilt may increase anxiety, interfere with sleep, and lead to depression [21,22]. The availability of clinical support and clear, simple communication can help reduce cancer patient confusion and distress [8].

A recent review showed that anxiety was more prevalent among cancer survivors than controls across all cancer types [23]. With survival rates for cancer improving with early detection and treatment, the failure to address psychological issues for cancer patients may reduce cancer survivor productivity and economic viability, thereby increasing the overall illness burden for patients, caregivers, and the health care system [24].

Several studies demonstrate correlations between levels of psychological distress in cancer patients and their families [25]. Cancer causes emotional distress in patents’ and their families’ lives. While the emphasis is on treating the physical symptoms of patients with cancer, families and caregivers who are also affected have limited support and/or restricted access to psychological treatment for their distress. This is a treatment gap that needs filling.

Provision of psychological services from diagnosis through treatment and rehabilitation may help cancer patients, families, and caregivers address the burden of psychological distress arising from their diagnosis. It is likely that significant and ongoing barriers to cancer patients accessing psychological supports have increased during the coronavirus disease (COVID-19) pandemic. Presently, cancer psychological services are either embedded in the cancer service itself, offered through community mental health services, or accessed privately. In Canada, these specialized services are usually located in urban areas, forming service clusters, thereby limiting access for patients, families, or caregivers who are remotely situated or self-isolating. In times of crisis, the mental health needs of cancer patients are anticipated to increase, but their service access may be restricted by medical service and public health constraints related to COVID-19. This creates a critical gap in service provision and care for these patients. To bridge this gap, there is a global call to embrace and adopt nontraditional means to service the mental health needs of vulnerable populations. Options include incorporating existing technologies such as telepsychiatry, internet-based cognitive behavior therapy (CBT) [26], and mobile messaging services [27]. Patients located in less populated or rural communities may find these services helpful in bridging the gap in care and provision of services.

The use of cellphones has become an integral part of daily living for a majority of the population. Mobile messaging services can be asynchronous, geographically independent, tailored, and contextualized for vulnerable groups. Text messaging is often economical (cents per message) or free for end users and does not require technical skills for use or expensive data plans; furthermore, almost 90% of Canadians own a smartphone [28]. Supportive text messages are associated with positive clinical outcomes in community settings, including a reduction in depressive symptoms and increased abstinence duration in alcohol use disorder [27,29]. In two user satisfaction surveys, over 80% of subscribers reported that a supportive text messaging program improved their mental health [27,30]. It is also a low-cost adjuvant to care that can be received at home when self-isolating and may be used to bridge the psychological treatment gap for medically vulnerable patients, including cancer patients, and particularly for those in remote or rural areas to improve service accessibility.

To help address the potential stressors and mental health difficulties that inevitably arise during emergencies, Alberta Health Services (AHS), in collaboration with six health foundations and the Department of Psychiatry at the University of Alberta, launched the Text4Hope-Cancer Care program. Text4Hope-Cancer Care is an evidence-based tool providing daily free CBT-based text messages for 3 months to individuals who self-subscribe to the program. It evolved from the pre-existing program infrastructure supporting the Text4Mood program, initially launched in January 2016 to support individuals in the aftermath of the 2016 Fort McMurray fires [27]. It was launched as Text4Hope [31] in March 2020 during the COVID-19 pandemic in order to improve access to psychological care in a cost-effective and timely manner to individuals who are self-isolating. For cancer patients in remote areas and for those self-isolating, there is an even greater urgency to support them, particularly when access to services is restricted. As an evidence-based bridge to achieve this, the Text4Hope-Cancer Care program is a distinct arm of the Text4Hope program. The messages are intended to help individuals identify and adjust negative thoughts, feelings, and behaviors arising from a cancer diagnosis and treatment as well as the COVID-19 pandemic.

### Objective

This protocol describes the implementation and evaluation of a low-cost, evidence-based, supportive text message service for cancer patients in Alberta. The objective of the project is to implement a self-subscribing daily supportive text message program to close the psychological treatment gap and reduce anxiety and depression related to cancer diagnosis. Research questions include:

1. Will the supportive text message program help to reduce anxiety and depressive symptoms among Albertan cancer patients and their caregivers?
2. What are the prevalence rates of anxiety and depressive symptoms in cancer patients and their caregivers who subscribe to the program?
3. What are the demographic correlates of anxiety and depressive symptoms in cancer patients and their caregivers who subscribe to the program?
4. Do demographic variables affect anxiety and depression amongst cancer patients and their caregivers when quarantined?
5. Will cancer patients and their caregivers be satisfied with receiving this method of care and support during the pandemic?

## Methods

### Evaluation Methodology and Measurement Plan

Patients diagnosed or receiving treatment for cancer and their caregivers can self-subscribe to receive daily supportive text messages for 3 months by texting the word “CancerCare” to 393939. The messages align with a cognitive behavioral framework and were developed by a clinical psychologist (MH), then reviewed and revised by a multidisciplinary team consisting of psychiatrists, mental health therapists, and counselors who work with cancer patients and their caregivers. Examples of the Text4Hope-Cancer Care messages are shown in Textbox 1.

**Text4Hope-Cancer Care messages.**

- Advocate for your needs using assertiveness. Assertiveness is being respectful to you and the other person. Be direct, non-aggressive, and specific.
- Do things you enjoy. These activities can remind you who you are, and take your mind off cancer for a while.
- Cancer affects the whole family. To help you and your family cope, try to: maximize quality time together, communicate, and create a schedule together.

Messages are preprogramed into a web-based text messaging service for online delivery at 12 PM each day. At the onset, and via the first message, respondents are welcomed to the service and invited to complete an online baseline survey capturing demographic information, COVID-19–related self-isolation/quarantine information, and self-reported responses on the Hospital Anxiety and Depression Scale (HADS) [32]. HADS is comprised of 14 questions, each of which has four answer options, with a total score calculated out of a possible 21 points. A total score of 0-7 is considered *Normal*, 8-10 is considered *Borderline Abnormal (Borderline Case)*, and 11-21 is considered *Abnormal (Case)*. HADS is the main measurement tool for the effectiveness of the Text4Hope-Cancer Care program.

Survey questions will be programed into Select Survey, an online survey tool, operated by AHS. Respondents will receive no incentives or inducements for participating in the program. Participation in the program is voluntary, and completion of the survey will not preclude receipt of subsequent supportive text messages. Subscribers may opt-out at any time by texting “Stop” to 393939. Survey responses will be stored on an AHS server and data will be exported and analyzed by the research team. Ethics approval has been granted by the University of Alberta Health Research Ethics Board (Pro00086163).

### Sample Size Considerations

Based on annual cancer diagnoses in Alberta, we expect about 20,000 patients with cancer and their caregivers to subscribe to the supportive text message program over the next year. Given a previous response rate of 21.7% for the Text4Mood survey [33], we anticipate around 4000 survey responses over the next 12 months.

### Outcome Measures

The primary outcome is changes in HADS scores at 6 and 12 weeks from baseline. The secondary outcomes are (1) interaction between primary outcomes and the demographic characteristics of subscribers and self-isolation/quarantine status during the pandemic; and (2) subscriber satisfaction and experience.

We will implement and evaluate the Text4Hope-Cancer Care program using the Reach, Effectiveness, Adoption, Implementation, and Maintenance (RE-AIM) Framework [34] and the Alberta Quality Matrix for Health [35]. Specifically, dimensions considered will include: Acceptability (subscriber satisfaction/experience), Accessibility (number of cancer patients and their caregivers that subscribe to and complete the program and ease of subscription to and utilization of Text4Hope-Cancer Care), Appropriateness (subscriber feedback related to how helpful the daily messages have been during the COVID-19 pandemic), and Effectiveness (6- and 12-week changes in the HADS). It may also be possible to examine Efficiency (cost avoidance and efficiencies through the reduced need for face-to-face counseling) and Safety (self-reports of decreased crisis and urgent service calls and decreased emergency medical services utilization rates).

### Hypotheses

Our hypotheses, based on previous research, are that: (1) higher rates of anxiety and depression will be reported among cancer patients and their caregivers who subscribe to the program compared to rates of these disorders in the general population; (2) specific risk factors will be found for the experience of anxiety and depression, such as female gender, self-isolation and quarantine status, and social determinants of health (eg, employment, housing); (3) the intervention will result in a 25% or greater reduction in anxiety and depressive symptoms (as measured by the HADS) at 6 and 12 weeks from baseline; (4) at least 80% of subscribers will express satisfaction with the supportive text message program and perceive the daily supportive text messages as contributing to their overall mental well-being.

### Data Analysis

We will evaluate the impact of Text4Hope-Cancer Care by analyzing the change of anxiety and depression symptoms at 6 and 12 weeks as reported on the HADS by subscribers. Data analysis will include standard use of parametric and nonparametric techniques (eg, within-subject general linear models), including multiple comparison type 1 error corrections. Power analysis with effect sizes based on Agyapong group research publications [27,29,36-39] indicates sufficient effect size for the expected Text4Hope-Cancer Care program subscriber sample size.

## Results

The first research stage, which was completed in April 2020, involved the creation and review of the supportive text messages and uploading of messages into a web-based text messaging service. The second stage, involving the launch of the Text4Hope-Cancer Care program, occurred in May 2020. The remainder of the project will focus on data analysis and reporting.

## Discussion

This research study proposes supportive text messaging to address a critical gap in psychological care for patients with cancer. The identification and management of emergent mental health issues for cancer patients has neither been addressed for Canadian nor other global jurisdictions during the COVID-19 pandemic. Similarly, the use of supported text messaging as a means of bridging the psychological treatment gap for cancer patients with mental health issues has not been studied previously. This study will offer support, independent of geographic location (urban, rural, or remote) or physical distancing, to cancer patients at a time when their mental health needs may be exacerbated by the COVID-19 pandemic. This approach will seek to ameliorate potential negative effects of self-isolation and social distancing measures in cancer patients and their access to psychological care in a cost-effective and responsive way. The study will make accessible and available psychological supports that would otherwise be inaccessible, risky, or significantly burdensome for patients to use, given their physical location, disease condition, or position on a waitlist. A significant portion of cancer patients do not take part in traditional psychological supports offered to them [40]. A plausible explanation for this may be an increased physical and mental tiredness, logistic difficulties getting to service locations, and geographic distance from the clinic, which may preclude their engagement in traditional, face-to-face, psychological support approaches. Using supportive text messaging to provide CBT-based psychological treatment at the individualized level may increase chances of participation and possibly improve engagement and outcome. This approach offers a nimble support system that may reach an individual at virtually any location and enables scalable access to a larger, geographically disparate population, fitting the demography of Canada and many other nations, including low and middle-income countries and the Asia-Pacific Economic Cooperation community.

A limitation of the study design is that it may selectively appeal to younger cancer patients and caregivers who have greater flexibility with the use of text messages, thereby producing selection bias. As well, phone service coverage may not be uniform throughout the province, with denser coverage for city populations, which may skew the findings toward a more urban population with a higher educational level. Finally, rates of anxiety and depression to be reported in our study will be based on a standardized self-rated scale (ie, the HADS) rather than clinical interviews using the Diagnostic and Statistical Manual for Mental Disorders, Fifth Edition (DSM-5).

## Abbreviations

AHS: Alberta Health Services
CBT: cognitive behavior therapy
COVID-19: coronavirus disease
DSM-5: Diagnostic and Statistical Manual for Mental Disorders, Fifth Edition
HADS: Hospital Anxiety and Depression Scale
RE-AIM: Reach, Effectiveness, Adoption, Implementation, and Maintenance
